# Regression hidden Markov modeling reveals heterogeneous gene expression regulation: a case study in mouse embryonic stem cells

**DOI:** 10.1186/1471-2164-15-360

**Published:** 2014-05-12

**Authors:** Yeonok Lee, Debashis Ghosh, Yu Zhang

**Affiliations:** Department of Statistics, Penn State University, University Park, PA 16802 USA; Department of Public Health Sciences, Penn State University, University Park, PA 16802 USA

**Keywords:** Regression hidden Markov model, Histone modification, Gene expression level, Mouse embryonic stem cell

## Abstract

**Background:**

Studies have shown the strong association between histone modification levels and gene expression levels. The detailed relationships between the two can vary substantially due to differential regulation, and hence a simple regression model may not be adequate. We apply a regression hidden Markov model (regHMM) to further investigate the potential multiple relationships between genes and histone methylation levels in mouse embryonic stem cells.

**Results:**

Seven histone methylation levels are used in the study. Averaged histone modifications over non-overlapping 200 bp windows on the range transcription starting site (TSS) ± 1 Kb are used as predictors, and in total 70 explanatory variables are generated. Based on regHMM results, genes segregated into two groups, referred to as State 1 and State 2, have distinct association strengths. Genes in State 1 are better explained by histone methylation levels with *R*^2^=.72 while those in State 2 have weaker association strength with *R*^2^=.38. The regression coefficients in the two states are not very different in magnitude except in the intercept,.25 and 1.15 for State 1 and State 2, respectively. We found specific GO categories that may be attributed to the different relationships. The GO categories more frequently observed in State 2 match those of housekeeping genes, such as cytoplasm, nucleus, and protein binding. In addition, the housekeeping gene expression levels are significantly less explained by histone methylation in mouse embryonic stem cells, which is consistent with the constitutive expression patterns that would be expected.

**Conclusion:**

Gene expression levels are not universally affected by histone methylation levels, and the relationships between the two differ by the gene functions. The expression levels of the genes that perform the most common housekeeping genes’ GO categories are less strongly associated with histone methylation levels. We suspect that additional biological factors may also be strongly associated with the gene expression levels in State 2. We discover that the effect of the presence of CpG island in TSS ± 1 Kb is larger in State 2.

**Electronic supplementary material:**

The online version of this article (doi:10.1186/1471-2164-15-360) contains supplementary material, which is available to authorized users.

## Background

As a part of an effort to understand the biological mechanism of gene regulation, epigenetic factors have been studied in conjunction with gene transcription [1]. Histone modifications are known as a major gene regulatory factor along with transcription factors [1, 2]. Studies suggest that chemically modified histones, such as methylated or acetylated, contribute to gene regulation by altering the DNA accessibility of transcription factors. When the transcription factors bind to a DNA promoter or enhancer region, they activate or enhance gene transcription, respectively [3]. There have been many studies for detecting the association between histone modifications and gene expression levels. In fact, a particular histone modification is associated with a specific function [4]. For example, H3K4 monomethylation (H3K4me1) is associated with gene enhancer activity. H3K4me3 and H3K27me3 promotes and represses gene expressions in mammalian stem cells, respectively.

Recently, in [5], the authors showed that gene expression levels can be predicted by histone modification levels using a linear model. As a continuation of the study, it is proposed in [6] that the association between histone modification levels and gene expression levels should be understood in the context of gene functions. They investigated the effects of histone modification levels in gene function groups and found that a combination of three histone modifications suffices to predict the gene expression levels in most of the gene functions. In [7], the authors verified the strong association between histone methylation levels and gene expression levels using support vector regression. Their studies are mainly aimed to verify the association between the two factors. Furthermore, a study to understand the effect of histone modification in conjunction of other regulatory elements was conducted in [8]. They identified the histone modification types that regulate gene expression levels in tissue/cell-specific genes.

Our study is built on the premise that the gene expression levels are strongly associated with histone modification levels but not universally predicted by a single relationship. Rather, the relationship varies across the genome. We look for multiple relationships between the gene expression levels and histone methylation by means of simple linear regression models in a hidden Markov model (HMM) framework. Based on the previous study results that there is a strong association between gene expression levels and the histone methylation levels, our further investigation finds state-dependent relationships between gene expression and histone methylation levels. An HMM is used under the spatial assumption that expressions of genes in a local cluster are influenced in a similar way by histone methylation levels.

HMMs have been widely applied in genetics and genomics [9, 10]. We apply a variant, called regression hidden Markov model (regHMM), that accounts for the relationship between the two sets of data. In our model, the response variable is the gene expression levels, and the explanatory variable is the histone methylation levels. A regHMM has been used in Engineering and Statistics [11, 12], but to our knowledge it has not been applied to study differential gene regulation. A regHMM can be considered a mixture of regression models with the Markov property in the hidden state, which is determined by the different associations between predictor and response variables in terms of regression coefficients and residual distributions. A regHMM can capture complex patterns in data while retaining the simple interpretation of standard linear regression models conditional on states. In addition, we implemented a distance-dependent transition probability feature in order to incorporate varying distances between genes. We compare the characteristics of the groups of genes splitted into two groups using the regHMM and find the biological differences.

## Results and discussion

**Figure 1:**
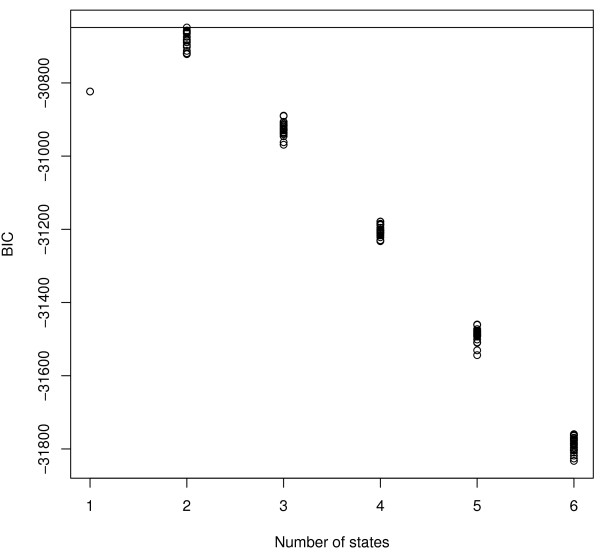
**Bayesian Information Criteria.** The plot shows the Bayesian Information Criteria values of a single-state regression and the regHMM of 2 to 6 states with 20 independent initial values. The maximum BIC occurs when the model has two states.

We applied a regHMM to understand relationships between the gene expression levels and histone methylation levels in mouse embrionic stem cells. We used 17020 gene expression levels as the response variable and the averages of seven histone methylation levels (H3K4me1, H3K4me2, H3K4me3, H3K9me3, H3K20me3, H3K27me3, H3K36me3) over 200 bp non-overlapping windows within the corresponding transcription starting sites (TSSs) ± 1 Kb as the explanatory variables. Based on the Bayesian Information Criterion (BIC) (Figure 1), we separated the genes into two groups of 10211 and 6809 genes using the Viterbi algorithm [13, 14]. We refer to the two groups as State 1 and State 2. See the Methods section for the regHMM model description, the data processing procedure, and BIC. We use the significance level *α*=.05 for statistical testing throughout the article.

### Characteristics of the two states

As a first step to explore the difference in two states, we performed a linear regression analysis for each state. The histone methylation levels explain about 72% of the gene expression variation in State 1 but only 38% of the variation in State 2. When regressing the gene expression levels on the fitted values using the two-state regHMM, 61.21% of the gene expression level variation is explained. In contrast, only 54.13% of the variation is explained using a single-state linear regression model to the entire data set. These results are summarized in Table 1.

**Table 1:** **The number of genes and** ***R*** ^**2**^ **for each state**

Condition	Number of genes	***R*** ^2^
State 1	10211	0.7133
State 2	6809	0.3842
Combined State 1 and State 2	17020	0.6121
All (a single regression)	17020	0.5413

**Figure 2:**
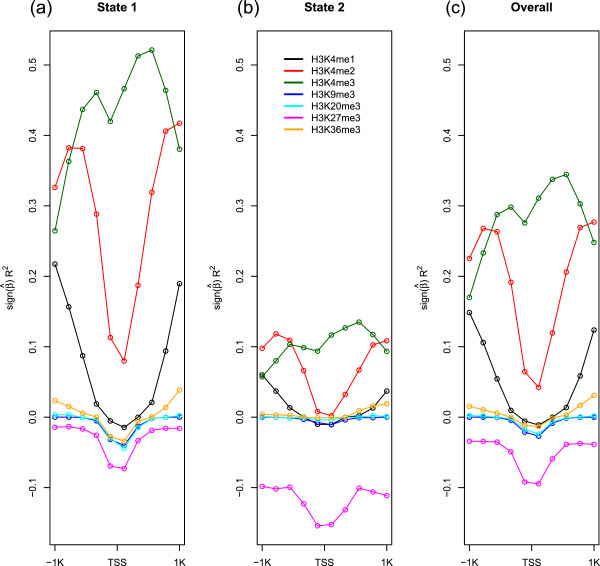
***R*** ^**2**^ **for individual predictor variables for each state and overall.** The *R* ^2^ multiplied by the sign of the regression coefficient  when gene expression levels are regressed on the individual predictor variables for each state are presented in **(a)** and **(b)** and for overall in **(c)**.

As the difference in *R*^2^ values in the two states was noticeable, we compared the proportion of variation explained by the individual variables for each state and presented them in Figure 2. For State 1, H3K4me2 and H3K4me3 are the two most significant factors for explaining the expression variation. Interestingly, H3K4me3 at TSS + 500 bp explained almost 50% of gene expression variation by itself in the plot (a) in Figure 2. For State 2, by contrast, only about 10% of the gene expression variation was explained by variables in H3K4me2, H3K4me3 and H3K27me3. The amount of gene expression variation explained by histone methylation levels is much smaller in State 2 than in State 1, except for H3K27me3.

**Figure 3:**
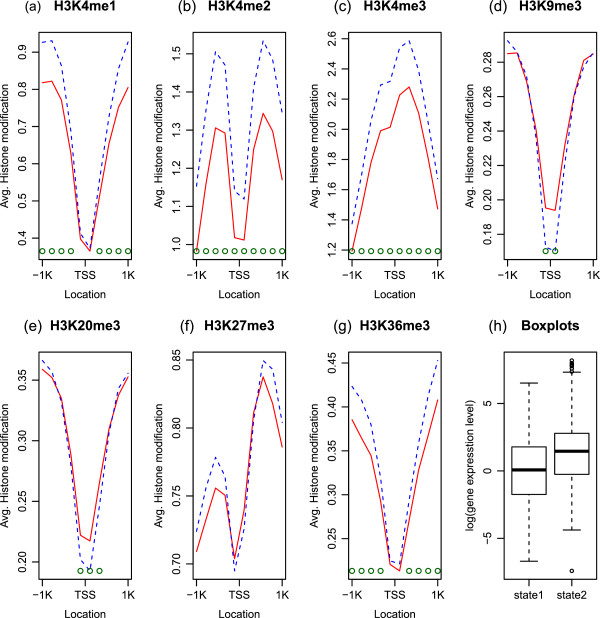
**Average histone methylation levels for each state.** The plots **(a)**–**(g)** show the average histone methylation levels in 200 bp non-overlapping windows on TSS ± 1 Kb region for each state. The red solid line and the blue dotted line represent State 1 and State 2, respectively. The green circles at the bottom indicate variables of which averages are statistically significantly different from 0 after the Bonferroni correction. The box plots in **(h)** show the gene expression levels for each state. The gene expression level averages are 0.09 and 1.29 and the medians are 0.07 and 1.46 in State 1 and State 2, respectively.

**Figure 4:**
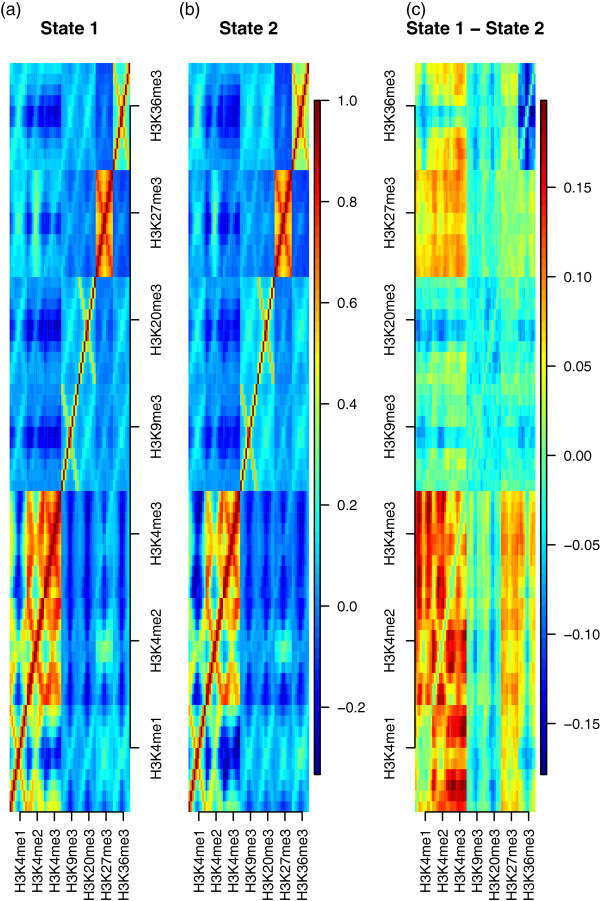
**Correlation of the histone methylation levels for each state and the difference.** The correlation of histone methylation levels for each state are in **(a)** and **(b)**. The difference of them (State 1 - State 2) is plotted in **(c)**.

We also investigated the average differences and correlation of histone methylation levels in the two states. The plots (a)–(g) in Figure 3 present the average histone methylation levels for each state. We use the raw-scale in order to show the histone modification patterns around TSS. The green circles at the bottom indicate explanatory variables of which the average differences of the two states are statistically significant after the Bonferroni correction. The average differences of the two states are more apparent in H3K4me1, H3K4me2, H3K4me3 (activators) and H3K36me3 than the rest. H3K27me3 is higher on average in State 2 relative to State 1, but the difference is not statistically significant with *α*=.05. The plot (h) in Figure 3 is the box plots of the gene expression levels for each state. Relative to State 1, the higher average gene expression levels in State 2 is attributable to the larger means of activators and enhancers (H3K4me1, H3K4me2, H3K4me3, and H3K36me3) and the smaller means of repressors (H3K9me3 and H3K20me3) in State 2. In Figure 4, the correlations of histone modification in the two states show similar trends in plots (a) and (b). The difference of the correlations between the two states in plot (c) in Figure 4 reveals the stronger correlations among H3K4me1, H3K4me2, and H3K4me3, and between the three features and H3K27me3 in State 1.

**Figure 5:**
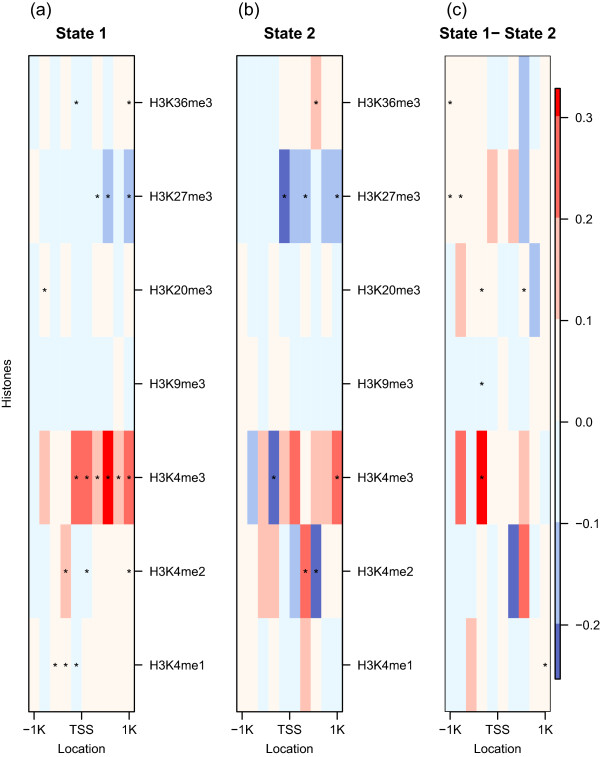
**Regression Coefficients.** Regression coefficients for each state are presented in **(a)** and **(b)** and the difference (State 1 - State 2) in **(c)**. The star marks in the plots **(a)** and **(b)** represent statistically significant coefficients and those in the plot **(c)** represent significantly different ones from each other after the Bonferroni correction. The intercepts (not shown) are.25 and 1.15 for State 1 and State 2, respectively.

Now we investigate the regression coefficients of all predictor variables jointly in each state. The condition numbers given a state are 20.44 and 21.00 for State 1 and State 2, respectively. See the Methods section for details on computation of condition number [15]. The coefficients of the two states are shown in the plots (a) and (b) and the difference are presented in the plot (c) in Figure 5. In both states, most of the variables for H3K4me3 are positively related with gene expression levels and negatively with most of the variables for H3K27me3. Three significant differences, out of eight, between two regression coefficients in the plot (c) in Figure 5 occur between TSS - 400 and TSS - 200. While most of the regression coefficients are not statistically different from each other, the intercepts,.25 and 1.15 for State 1 and State 2, respectively, are notably different (excluded in Figure 5 as the magnitude of the intercept in State 2 is much larger than the remaining coefficients). Recall that an intercept is interpreted as the average gene expression level when all the histone modification levels are 0. The larger intercept with the larger gene expression averages in State 2, yet the weaker association with histone methylation levels in State 2, suggests that genes in State 2 may be further affected by other biological factors.

It is intriguing that the gene expression levels in State 2 are larger on average while having weaker association with the histone methylation levels. We suspected that the histone methylation levels in the wider region may better explain the variation of gene expression levels, so we repeated the study over an extended region (TSS ± 4 Kb). However, the study results are similar to those on TSS ± 1Kb and they are summarized in Additional file 1. A model with two states is chosen via BIC (Additional file 1: Figure A1) and the state with larger gene expression levels on average has the weaker association with histone methylation levels (Additional file 1: Table A2 and Figure A2).

### Biological differences in two states

We considered a collection of the embryonic stem cell-specific genes selected against genes in differentiated cells [16] and examined the differences in the two states. We used 342 embryonic stem cell-specific genes that are both in our study and in the list of 543 genes (*FDR*<.025) in [16]. There are 161 and 181 genes in each state. The difference in *R*^2^ (*R*^2^=.7288 and *R*^2^=.5148 for State 1 and State 2, respectively) is statistically significant with *p*-value =.009. See Simulation-based test of the difference in *R*^2^ in Methods section. Due to multicollinearity issue in the predictor variables for stem cell-specific genes (the condition numbers were 35.61 and 28.92 for State 1 and State 2, respectively), we did not compare the regression coefficients from stem cell-specific genes in each state.

**Figure 6:**
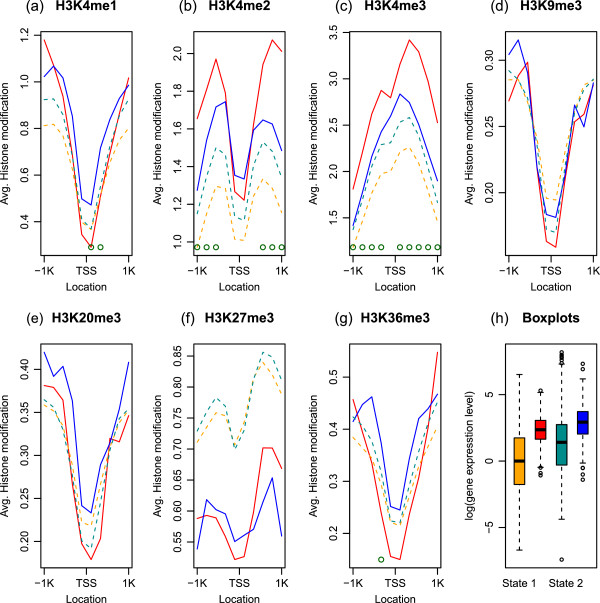
**Average histone methylation levels of non-stem cell-specific and stem cell-specific genes for each state.** The average histone methylation levels of the non-embryonic stem cell-specific and the embryonic stem cell-specific genes in State 1 are represented by an orange and a red curves, respectively, and those in State 2 are represented in a cyan and a blue curves, respectively, in **(a)**–**(g)**. Box plots in **(h)** represent the non-embryonic stem cell-specific and the embryonic stem cell-specific gene expression levels for each state. The green circles at the bottom indicate variables of which average histone methylation levels of the stem cell-specific genes are statistically significantly different from 0 after the Bonferroni correction.

We presented the average histone methylation levels of the non-stem and the stem cell-specific genes for each state in Figure 6: the stem cell-specific genes (red and blue for State 1 and State 2, respectively) and the non-stem cell-specific genes (orange and cyan for State 1 and State 2, respectively). The green circles at the bottom of the plots (a)–(g) in Figure 6 indicate variables of which the difference in the average histone methylation levels of stem cell-specific genes in two states are statistically significant after the Bonferroni correction. For H3K4me2 and H3K4me3, the average histone methylation levels of stem cell-specific genes in State 1 shifted much larger than that of State 2. This may explain the larger increase (by 2.2) in the average stem cell-specific gene expression levels in State 1, compared to the increase (by 1.58) in State 2. The plot (h) in Figure 6 shows the box plots of expression levels of the stem cell-specific and the non-stem cell-specific genes for each state.

The average H3K27me3 levels for the stem cell-specific genes are much smaller than the average of the rest of the genes for each state, which is consistent with the repressing role of H3K27me3 [17]. It is interesting that the average histone methylation levels of H3K20me3 and H3K36me3 shifted in the opposite directions in most of the TSS ± 1 Kb range. The average histone methylation levels for the stem cell-specific genes increased in State 2 but decreased in State 1. We also observed such changes over TSS ± 200 bp in H3K4me1 and H3K9me3. These changes toward the opposite directions in the average histone methylation levels, while the consistent increase in expression levels for the stem cell-specific genes in both states, support our claim that the relationship between gene expression levels and histone methylation levels is not universal.

We tried to understand the difference in the two states by including CpG islands, which has been found to be strongly indicative of promoter activities [18]. Also, CpG islands influence the chromatin structure [19]. We found that genes in State 2 are more frequently overlapped with CpG islands on the TSS ± 1 Kb regions than genes in State 1 (*p*-value <2.2·10^-16^, see Table 2). To study the effect of CpG islands in TSS ± 1 Kb on the gene expression levels, we defined a new variable CpG indicating the presence of CpG islands overlapping with the TSS ± 1 Kb region (1: presence, 0: absence). We included the CpG variable in addition to the 70 explanatory variables and performed regression analyses in each state. The CpG variable improved the model fit with *p*-values <2.2·10^-16^ for both states (*R*^2^=0.7182 and *R*^2^=0.3938 for State 1 and State 2, increased by.0049 and.0096, respectively, from the original models). The intercepts changed from 0.25 and 1.15 to -0.05 and 0.73 in State 1 and State 2, respectively. The regression coefficients of the CpG variable were.48 and.64, respectively, where the effect of CpG in State 2 was statistically significantly larger than that of State 1 (*p*-value = 0.0252).

**Table 2:** **Genes whose TSS ± 1 Kb overlaps with CpG island**

	State 1	State 2
No overlapping with CpG Island	4154 (41%)	2051 (30%)
TSS ± 1 Kb overlaps CpG Island	6057 (59%)	4758 (70%)

We also studied if genes that include a TATA motif (TATA-containing genes) are enriched in one of the state. There are 1829 TATA-containing genes on TSS-100 bp to TSS window. Among these, 1200 and 629 are in State 1 and State 2, respectively. There is statistically significant TATA motif enrichment in State 1 (*p*-value = 2.42·10^-7^). To study the effect of presence of TATA box in 100 bp downstream from TSS on the gene expression levels, we defined a new variable TATA indicating the presence of TATA-box (1: presence, 0: absence) and added to the 70 explanatory variables to perform regression analyses in each state. The TATA variable improved the model fit with *p*-values =1.75·10^-5^ for State 1 (*R*^2^=0.7138, increased by 0.0005 from the original models) but not for State 2 with *p*-value =0.1561. The TATA-containing gene enrichment in State 1 seem to be supported by the study results that TATA-less genes in human are frequently involved in housekeeping functions while TATA-containing genes are highly regulated [20], which we will discuss shortly.

### Gene Ontology analysis

In [6], they use a combination of three histone modifications to explain the gene expression variation among genes with the same functional annotations. Similarly, to explore the attribution of gene functions to the expression variation, we conducted a Gene Ontology-based (GO-based) enrichment analysis [21, 22]. Genes can have multiple GO annotations, not only because genes conduct various functions in cells but also because GO annotations are hierarchical. There are many genes without GO annotations, too. For State 1 and State 2, we found 2077 and 1217 genes without any GO annotations, respectively.

We calculated the gene frequencies in the same GO annotations in each state. If there are more than 10 genes in the GO category in each state, we test if the proportions are significantly different in the two states. Such GO annotations (after the Bonferroni correction) are listed in an increasing order of *p*-values in Additional file 2: Table A1. The gene functions enriched in State 1 are related to cellular signal transduction functions. The GO annotations enriched in State 2 show a broad range of gene activities. We noticed that the top four GO annotations in State 2 in Additional file 2: Table A1 match to the most frequent housekeeping gene functions (or GO categories) in Table 3. See the Housekeeping genes in Methods section for the details. There are 192 and 239 housekeeping genes in State 1 and State 2, respectively (total 431), and housekeeping genes are more frequent in State 2 (*p*-value = 4.693·10^-11^). The housekeeping gene expression variation explained by histone methylation levels in State 1 and State 2 are *R*^2^=.5104 and *R*^2^=.4066, respectively. The *R*^2^ difference is not statistically significant (*p*-value =.298). See Simulation-based test of the difference in *R*^2^ in Methods section.

**Table 3:** **The six most common GO annotation among housekeeping genes**

GO annotation	Count	Proportion (Count/528)
Cytoplasm (GO:0005737)	244	0.46
Nucleus (GO:0005634)	228	0.43
Protein binding (GO:0005515)	137	0.26
Membrane (GO:0016020)	123	0.23
Mitochondrion (GO:0005739)	97	0.18
Nucleotide binding (GO:0000166)	92	0.17

Additionally, the two *R*^2^ from the housekeeping genes for each state are significantly smaller than the *R*^2^ of random selection of the same number of genes (both *p*-values <0.001). When a set of random genes of size 192 is regressed on the corresponding histone methylation levels, the minimum *R*^2^ is.5351 (with 1000 repetitions). Likewise, when 239 random genes were regressed, the minimum *R*^2^ is.5245 (with 1000 repetitions). The *R*^2^ of a single regression of all housekeeping genes on the 70 histone modification levels is only.2677.

From this study, we conclude that housekeeping gene expression levels are not as well explained as other genes by the seven histone methylation levels in mouse embryonic stem cells using a linear regression model. This may be due to the fact that genes required to maintain basic cellular functions may be regulated by other biological factors such as DNA methylation. This is also consistent with the study results that housekeeping functions are represented in high CpG class [23]. Among 431 housekeeping genes, 407 (94.43%) have CpG island within 1 Kb from their TSSs.

We explored the gene expression level variations with the same GO annotations in Additional file 2: Table A1. The last column shows the gene expression variation explained by the histone modification levels in the corresponding GO annotation using a linear regression model. For example, the gene expression variation explained by histone methylation in G-protein coupled receptor signaling pathway is *R*^2^=.5573 and the simulation-based *p*-value is 0.121 (See Simulation-based test of a large or small *R*^2^ in Methods section). The *R*^2^ and the test probability are shown in Additional file 2: Table A1, where the blank cells are due to the small number of genes in the GO annotation to conduct a regression analysis. The last three GO annotations in State 1 and ruffle in State 2 are not studied for this reason.

In State 1, receptor activity, integral to membrane, and signal transduction are the only GO annotations in which the gene expression levels are better explained by histone methylation levels (than random selection of genes). The GO annotations more frequent in State 2 in Additional file 2: Table A1 show consistently lower *R*^2^ than random, except for perinuclear region of cytoplasm.

### Prediction

We finally examine the prediction performance of regHMM using five-fold cross-validation. We divided the data into training and test data sets in size 13616 (80%) and 3404 (20%), respectively. We applied the regHMM to the training data and evaluated how well the trained model can predict the gene expression levels in the test data set. We repeated the study 15 times and the results were similar to each other; we thus present one such result. The training data set is segregated into two states: one has *R*^2^=.7488 (8062 genes in State 1) and another has *R*^2^=.3843 (5554 genes in State 2). In conventional hidden Markov models, the hidden states of test data are predicted using both the information the data carry and the transition probabilities. Meanwhile, our interest is in predicting the missing response values in test data. To predict the gene expression levels, we use the distinctive histone methylation profile information in the two states and specified the states in the test data set by the following approach. Let the histone methylation levels of the test data be *z*_1_,⋯,*z*_*K*_, where *K*=3404 denotes the sample size of the test data. We evaluated the probability that *z*_*i*_ belongs to state *m*, *p*_*m*_(*z*_*i*_), assuming that *z*_*i*_ follows a multivariate Normal distribution  for *m*=1,2, where  denotes the sample mean and *V*_*m*_ denotes the sample variance of histone methylation levels in the training data in State *m*. Let the proportion of State 1 be *ϕ* and the proportion of State 2 be 1-*ϕ*. We assigned a gene in the test data into State 1 if *ϕp*_1_(*z*_*i*_)>(1-*ϕ*)*p*_2_(*z*_*i*_) and State 2 otherwise. The test data of 3404 genes were divided into 1521 genes in State 1 and 1883 genes in State 2. When the true gene expression level is regressed on the predicted gene expression level in State 1, we obtained *R*^2^=0.6065. When a single regression model is applied to the test data set, the *R*^2^=0.5234. For the genes assigned to State 2 in the test data, we obtained *R*^2^=.3405. Note that knowing that genes in State 2 are not well explained by the histone methylation levels, it will not be meaningful to try to predict the expression levels in State 2.

### Discussion

We looked into the possibility that the models with a larger number of states may lead to more interesting biological interpretation. For example, we searched for more specific classifications of stemness or pluripotency genes in three-state and four-state models. We found similar trends in two-state model. Overall, we found exactly 2 out of 3 (or 4) states in the 3-state (or 4-state) model that have common GO annotation enrichment with those observed in the 2 state model, whereas the rest states do not have significant GO annotation enrichment. In addition, the states with the lowest *R*^2^ show the highest average and median gene expression levels. We suspect that it is in part due to the facts that the genes that are annotated with stemness or pluripotency may not have strong association trend with histone methylation levels. The results are shown in Additional file 3.

About 22% of high CpG promoters in embryonic stem cells have bivalent domain [24], on which both H3K4me3 and H3K27me3 are catalysed. We obtained a list of genes on such domain in ESC [24]. Among the 13739 common RNA accessions, there are 1500 and 918 accessions out of 8319 (State 1) and 5420 (State 2), respectively. We tested the enrichment using the Chi-square test and the two states does not distinguish the genes on bivalent or non-bivalent domains (*p*-value = 0.1047). The model we proposed in this manuscript groups the genes by the relationships between the seven histone methylation levels and the gene expression levels. It is noteworthy that the study results are specific to data we applied, therefore it is cell-line specific.

The H3K4me1 mark is known as enhancer and affects the gene expression in distance. The median distance to the closest gene from the peak of H3K4me1 is 26739 in ES cell [25] and the minimum is 1008. Suspecting that there may not be no effect of H3K4me1 near TSS, we compared the model with and without H3K4me1. There is statistically significant effect of H3K4me1 (*p*-value <2.2×10^-16^). Despite the statistical significance, the changes in *R*^2^ is fairly minor (from *R*^2^=.5323 without H3K4me1 to *R*^2^=.5413 with H3K4me1). Also we see statistically significantly differences in averages of H3K4me1 in two states on the bins at least 200 bp away from TSS.

In an effort to improve the correlation for State 2, we considered including the two acetylation levels (H3K9ac and H3K27ac). When we included these two additionally, the *R*^2^ in State 1 and State 2 increased to.7554 and.479 (from.7133 and.3842, respectively) using the states obtained using the seven histone methylation levels. Further investigation is needed to carefully evaluate the effects of acetylation on expression on top of methylation, which is beyond the scope of this manuscript.

## Conclusion

It has been known that the gene expression levels are highly associated with the histone methylation levels [7]. Studies such as [6] and [8] have also tried to understand gene regulation in specific conditions. Yet, our understanding of the biological dynamics of epigenetic marks on gene expression remains limited. To better understand the biological mechanism in gene regulation, we investigated the potential multiple relationships between gene expression levels and histone methylation levels around TSS ± 1 Kb in mouse embryonic stem cells. The genes are categorized into two groups, and the gene expression levels are better explained by histone methylation levels in one group (State 1) than in another (State 2). The gene expression levels are higher on average in State 2 but the association strength with histone methylation levels is weaker. We suspect that the genes in State 2 may have different biological dynamics than genes in State 1 in addition to the association of histone methylation and gene expression levels. This is supported by that observation that the intercept in State 2 is about four-fold larger than the intercept in State 1. We also investigated possible attributions of the presence of CpG islands and the gene functions in the embryonic stem cells that may be related to distinct association strengths of the two states. The presence of CpG island in TSS ± 1 Kb has a significantly larger effect on gene expression levels in State 2. By comparing the GO annotation frequencies in each state, we found that gene expression may respond differently to the underlying histone methylation depending on gene functions. Genes related to receptor activity, integral to membrane, and signal transduction are more frequent in State 1 and have much stronger association with histone methylation levels. In comparison, genes with GO annotations cytoplasm, nucleus, protein binding, and more are frequent in State 2 and are not as strongly associated with histone methylation levels than randomly selected genes. The gene functions or GO categories enriched in State 2 tend to correlate with those of housekeeping genes. This leads us to test the association between the housekeeping genes and histone methylation levels, and find that the housekeeping genes are not as well-explained as random genes by histone methylation levels on TSS ± 1 Kb region. By studying stem cell-specific gene expressions, we further found interesting changes in the average histone methylation levels in the two states. The average expression levels of the stem cell-specific genes are higher than the rest of the genes in both states while the averages of H3K20me3 and H3K36me3 changed in opposite directions in the two states.

Using the regHMM model, we found two significantly different relationships between histone methylation levels and gene expression levels. The DNA structure around the genes such as CpG islands and the functions of genes in mouse embryonic stem cells also explained in part how the two states were different. However, these results are far from comprehensive understanding towards the complex mechanisms in gene regulation in large. It will be meaningful to further investigate the effects of histone modification on the changes of gene expression levels over time including additional regulation factors into consideration. The current study serves as a proof of concept in the power of data integration in order to further advance biological insights.

## Methods

### Regression hidden Markov model

A regHMM is a variation of a hidden Markov model in which the emission probability accounts for the relationship between two variables. The regHMM can be considered as a mixture of regression models with the Markov property in the latent state. The model can incorporate complex structures of the data with a mixture of simple regression models and it pertains the simplicity and interpretability of a simple regression model. Particularly for the data we analyzed, regHMM incorporates continuous dependent variables by a regression model as opposed to a logistic regression model for binary data and regHMM further incorporates the potential correlation due to genomic proximity [26] using the hidden Markov model framework.

Let *t*_*i*_ denote time or location at where the data is observed with *t*∈{*t*_1_,⋯*t*_*T*_} (*t*_1_<⋯<*t*_*T*_). Let *q*=(*q*_1_,⋯,*q*_*T*_) denote a vector of unknown states. Given the number of states *M*, let *s*_*i*_ denote the *i*th state *i*=1,⋯,*M*. An HMM with non-homogeneous transition probabilities can be structured by specifying the number of states *M*, the initial state probability *π*, the transition probability *A*_*t*_ at time *t*, *t*∈{*t*_1_,⋯*t*_*T*_}, and the emission probability density. The initial state probability is denoted by *π*=(*π*_1_,⋯,*π*_*M*_), where *π*_*i*_=*P*(*q*_1_=*s*_*i*_). The transition probability from state *s*_*i*_ at the (*t*-1)th time to state *j* at the *t*th time is denoted by *a*_*ijt*_=*P*(*q*_*t*_=*s*_*j*_| *q*_*t*-1_=*s*_*i*_,*d*_*t*_), where *d*_*t*_ denotes the distance between the *t*th time and the (*t*-1)th time for *i* and *j*=1,⋯*M* and *t*∈{*t*_1_,⋯,*t*_*T*_}. The emission probability density is denoted by *b*_*i*_(*O*_*t*_)=*p*(*O*_*t*_|*q*_*t*_=*s*_*i*_,*λ*), where *λ* is a collection of emission probability density parameters for *i*=1,⋯,*M*.

With an assumption on the distribution of the latent variables, the Baum-Welch algorithm [27], equivalent to the expectation maximization algorithm [28] for HMMs, finds the maximizer of the expected log-likelihood over the latent variables. The joint distribution *P*(*O*,*q*|*λ*) is


We consider a regHMM that incorporates two sets of data. Let *O*_*t*_=(*x*_*t*_,*y*_*t*_) denote the observation at the *t*th time, where  and  denote the response and the explanatory variables, respectively, and *d* and *p* denote their dimensions, respectively. We denote the emission probability given the state by *p*(*O*|*q*,*λ*)=*p*(*y*|*x*,*q*,*λ*)·*p*(*x*|*q*,*λ*). When *P*(*y*|*x*,*q*)=1, the model reduces to a usual hidden Markov model. When *p*(*x*|*q*,*λ*)=1, the emission probability depends only on the relationship between the two variables, which then reduces the model to the regHMM we applied in this study. Conditional on the states, we assume that the explanatory variables are linearly related to the response variables and the emission probability follows Normal distributions:


where *β*_*i*_ denotes the regression coefficient vector and *Ω*_*i*_ denotes the variance for *i*=1,⋯,*M*.

If the observations are unequally spaced, the transition probability from one state to another may be affected by the distance between two adjacent observations. We implemented the following transition probability [10]:
1

where *D* denotes a predetermined constant. As *D* gets larger, the chance of staying in the same state in the adjacent observations becomes larger. The probabilities *p*_*ij*_ are calculated by Baum-Welch algorithm. In [29], the study only included genes at least 4 Kb away from each other. In our study, instead, we included all the genes regardless of their distances, and we allowed the potential spatial relation between genes using the non-homogeneous transition probability, with *D*=4000 in Eq (1). The program MRHMMs [30] is used for analysis.

**Figure 7:**
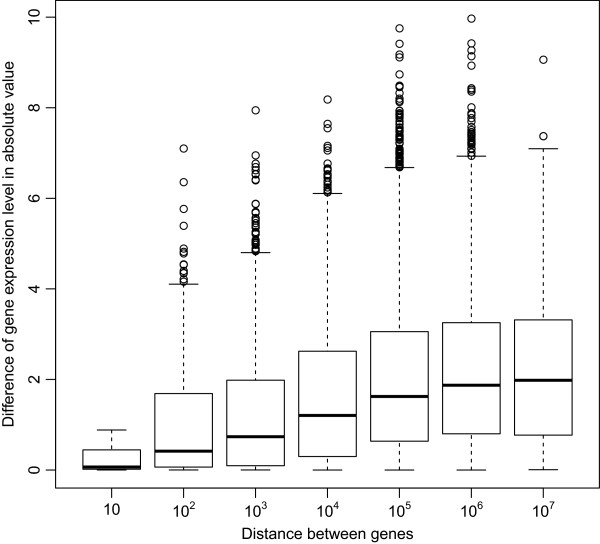
**Distance between genes and gene expression level differences.** Box plots of the expression level differences in two adjacent genes when the distance 10^*k*-1^≤dist<10^*k*^ is represented by 10^*k*^, for *k*=1,⋯,7.

We assumed the Markov property based on the idea that genes in proximity may be related to their similar expression levels [26]. In our data set, the gene expression level difference of the two adjacent genes is positively correlated with the distances between them. Figure 7 shows the box plots of gene expression level differences in absolute value for the distance between the genes. Additionally, based on BIC, a mixture of regression models in hidden Markov framework works better (BIC = -30702.77) than a mixture of regression models without the Markov property (BIC = -30752.93).

The Viterbi algorithm [13, 14] is used to find the most likely sequence of hidden states. The number of states are selected by Bayesian Information Criterion (BIC). According to [31], BIC performs well even in the presence of the Markov property.

### Data

We used seven histone methylation levels (H3K4me1, H3K4me2, H3K4me3, H3K9me3, H3K20me3, H3K27me3, H3K36me3) in mouse embryonic stem cells [24, 32] as the explanatory variables and gene expression levels as the response variable. The gene expression levels are calculated by [7] using the mapped RNA-seq reads for mouse ESC from [33] according to the reads per kilobase of exon per million (RPKM) mapped sequence reads defined in [34]. The gene expression level data is contributed by the authors of [33].

We focused our study on the TSS ± 1 Kb regions, at where the signals showed the most dramatic changes. We took the average of the histone methylation levels in 200 bp non-overlapping sliding windows over non-masked regions. As a result, we obtained 10 explanatory variables for each of the 7 histone methylation data and 70 explanatory variables in total. We standardized the data to have marginal normal distributions by mapping the quantiles of data to a standard normal quantile. To break ties, we added a small disturbance to the original data from *N*(0,0.01).

For the 25640 gene expression levels in [7], we assigned the corresponding RNA accessions (of the format NM_123456 or NM_123456789). Their TSS positions are obtained in mm8 from UCSC genome browser [35]. We excluded 174 genes that were not listed as RefSeq in UCSC genome browser and additional 44 genes that did not match to the strand directions listed in the UCSC genome browser. We excluded 5712 genes whose corresponding explanatory variables contained at least one masked position. We further removed 167 genes whose gene expression levels were 0. This data process is summarized in Table 4.

**Table 4:** **Data processing procedure**

Condition	Number in the condition	Number left
All	25640	25640
No match with refGene	174	25426
No match in strand direction	44	25382
Masked regions	5712	19670
Zero expression level	167	19503
Multiple expression levels with the same TSS	2483	17020

The acetylated histone levels (signal) are obtained from GSM1000147 and GSM1000099.

### Condition number

Let *X* be a *T*×*p* matrix, where *T* denotes the sample size and *p* denotes the number of explanatory variables. Due to the correlation between explanatory variables within the 7 histone methylation levels, there is a potential multicollinearity problem. We used the condition number, the square root of the ratio between the largest and the smallest eigenvalues of *X*^′^*X*, to detect multicollinearity. If the value is less than the cutoff 30 [15], we consider it as no multicollinearity. The condition number of *X* in our data was about 20.

### Housekeeping genes

We used the list of the housekeeping genes defined in [36]. Among the 622 genes on the website [37], we used housekeeping genes listed as either Known Genes or RefGenes in UCSC genome browser [35] to get the corresponding RNA accessions, and we obtained 528 genes. Genes categorized as either current or old symbol on [22] were used to build Table 3.

### Simulation-based test of the difference in *R*^2^

To test if there is a statistically significant difference in *R*^2^ between stem cell-specific and other genes:

1. Randomly sample 161 genes (the number of stem cell-specific genes in State 1) from the stem cell-specific genes and evaluate *R*^2^
2. Use the rest of stem cell-specific genes to evaluate another *R*^2^
3. Repeat the procedure 1000 times and count the number of procedures in which the difference between the two *R*^2^s is larger than the observed difference.

To test if there is statistically significant difference in *R*^2^ between the housekeeping genes in State 1 and State 2:

1. Randomly sample 192 housekeeping genes and evaluate *R*^2^
2. Find another *R*^2^ using the rest of the housekeeping genes
3. Repeat the procedure 1000 times and count the number of procedures in which the *R*^2^ difference is larger than the observed *R*^2^ difference.

### Simulation-based test of a large or small *R*^2^

To test if this *R*^2^ is significantly large or small in State 1 and State 2, respectively, in a specific GO annotation:

1. Randomly select the same number of genes in the GO annotation group. As an example, 671 (549 + 122) in G-protein coupled receptor signaling pathway
2. Evaluate the *R*^2^
3. Repeat 1000 times and count the number of procedures in which the *R*^2^ is smaller than the observed *R*^2^.

### Bayesian Information Criteria

The number of regression relationships is unknown and is selected based on the Bayesian Information Criteria [38]. Let *M* denote the number of states. Let *L*_*M*_(*O*,*q*) and *N*_*M*_ denote the log-likelihood and the number of parameters for model with the number of states *M*, respectively. The model that has the maximum of *L*_*M*_(*O*,*q*)-.5·*N*_*M*_ ln(*T*) is chosen as the best model.

## Electronic supplementary material

Additional file 1: **Analysis results on the region TSS ± 4 Kb.** Additional file 1 contains the analysis results using regHMM over the TSS ± 4 Kb region. (PDF 652 KB)

Additional file 2: **A table of the more frequent GO annotations in each state.** Additional file 2 contains a table describing the more frequent GO annotations in each state. The GO annotations are in bold text if the genes in the same GO annotation are better (or worse) explained by histone methylation levels in State 1 (State 2) than random genes in the annotation group of size equal to the number of genes. (PDF 39 KB)

Additional file 3: **Analysis results for 3-state and 4-state models.** Additinal file 3 contains a table describing the number of genes in each state and *R* ^2^, GO analysis results, and average histone methylation levels for 3-state and 4-state models. (PDF 123 KB)

## Abbreviations

regHMM: Regression hidden Markov model
TSS: Transcription starting site
H3K4me1: Monomethylated histone H3 lysine 4
H3K4me2: Dimethylated histone H3 lysine 4
H3K4me3: Trimethylated histone H3 lysine 4
H3K9me3: Trimethylated histone H3 lysine 9
H3K20me3: Trimethylated histone H3 lysine 20
H3K27me3: Trimethylated histone H3 lysine 27
H3K36me3: Trimethylated histone H3 lysine 36
BIC: Bayesian Information Criteria
GO: Gene Ontology
RPKM: Reads per kilobase of exon per million.
