# Supplementary material for: Regression hidden Markov modeling reveals heterogeneous gene expression regulation: a case study in mouse embryonic stem cells
Source: BMC Genomics. 2014 May 12;15(1):360. doi: 10.1186/1471-2164-15-360 (PMC4144088; doi:10.1186/1471-2164-15-360)
Supplement: Supplementary file 1 — Additional file 1: Analysis results on the region TSS ± 4 Kb. Additional file 1 contains the analysis results using regHMM over the TSS ± 4 Kb region. (PDF 652 KB) [file 12864_2013_6176_MOESM1_ESM.pdf]

SUPPLIMENTAL FILE 1

Supplemental file 1: Analysis results over the study region TSS ± 4Kb

Yeonok Lee*†, Debashis Ghosh† and Yu Zhang†

*Correspondence: yul26@psu.edu
Department of Statistics, Penn State University, University Park, USA
Full list of author information is available at the end of the article
†Equal contributor

We extend the study region to TSS ± 4Kb. We use the averages of histone modification levels over 400 bp non-overlapping windows as the explanatory variables. Each histone modification has 20 variables and in total we have 140 explanatory variables. As the range of study gets wider, it includes more non-masked regions and we have smaller sample size (14643) for the study. See Table 1 for the detailed data process. Based on the BIC, the model with two states is selected in Figure 1. It is presented in Table 2 that the number of genes the corresponding R^2 for the given condition. The $R^2 = 0.547$ when applied a single linear regression model improved to 0.6339 when using two linear regression models in the hidden Markov model framework. The R^2 for each state are similar to the results of the study over TSS ± 1Kb: in one state the R^2 is much larger than another. Figure 2 shows the R^2 values when the gene expression levels are regressed on the individual predictor variables. Again, the individual predictors for H3K4me3 explain more than 50% of gene expression variation near TSS. The plots (a) and (b) in Figure 5 show the regression coefficients the difference is in plot (c). The correlation of the histone modification levels for each state are shown in plots (a) and (b) in Figure 4 and the difference of the correlations in (c). They also show similar trend as in the main text. Figure 3 shows the average gene expression levels in each state in (a)-(g). The statistical differences in the averages for each state occur on H3K4me1, H3K4me2, H3K4me3, and H3K36me3. The box plot of the gene expression levels in (h) shows that gene expression levels in State 2 is larger than State 1.

Table A1 Data process procedures for TSS ± 4Kb

Condition	Number in the condition	Number left
All	25640	25640
No match with refGene	174	25426
No match in strand direction	44	25382
Masked regions	8506	16876
Zero expression level	156	16720
Multiple expression levels with the same TSS	2077	14643

Table A2 The number of genes and R^2 in each state for TSS ± 4Kb

Condition	Number of elements	R^2
State 1	9460	0.7075
State 2	5157	0.4260
Combined State 1 and State 2	14643	0.6339
All (a single regression)	14643	0.5470

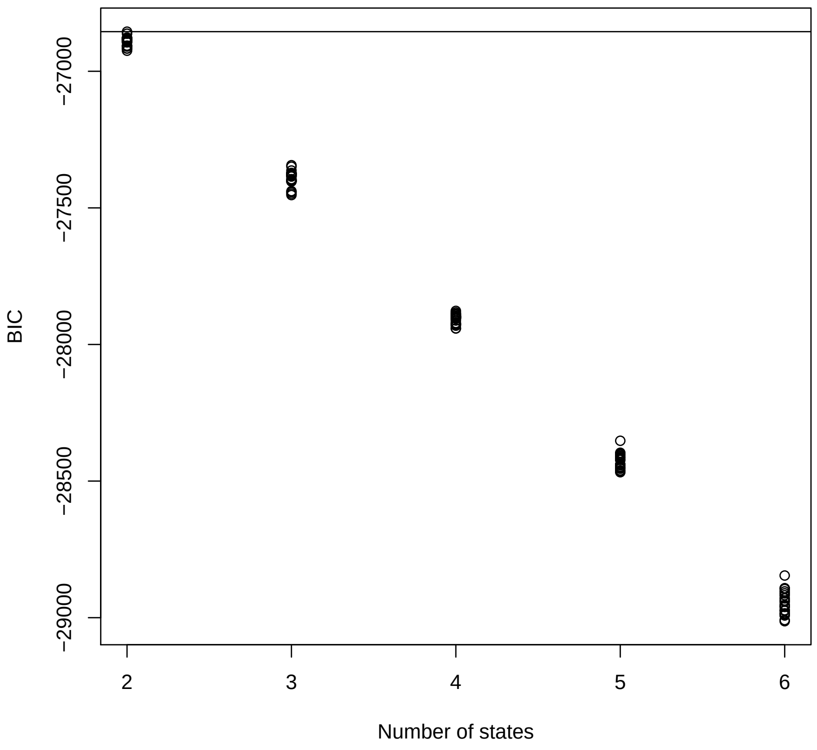

Figure A1 Bayesian Information Criterion over TSS $\pm$ 4Kb The plot shows the Bayesian Information Criteria values of rHMMs of 2 to 6 states with 20 initial values. The maximum BIC occurs when the model has two states ($M = 2$).

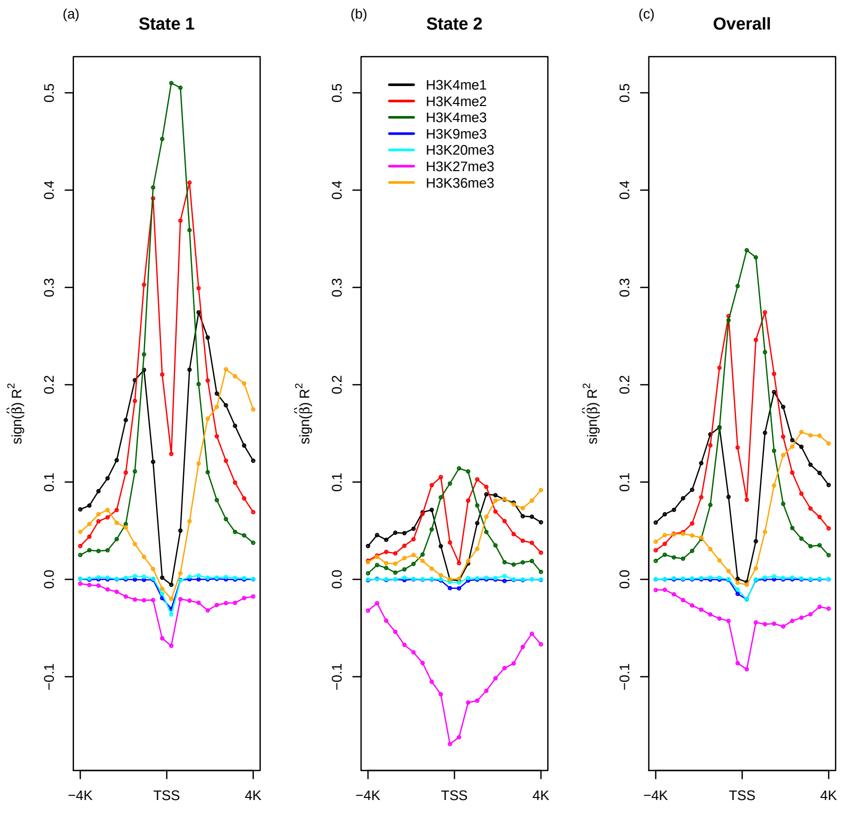

Figure A2 R^2 **for individual predictor variables for each state and overall on TSS** $\pm$ **4Kb** The R^2 multiplied by the sign of the regression coefficient $\hat{\beta}$ when gene expression levels are regressed on the individual predictor variables for each state are presented in (a) and (b) and for overall in (c).

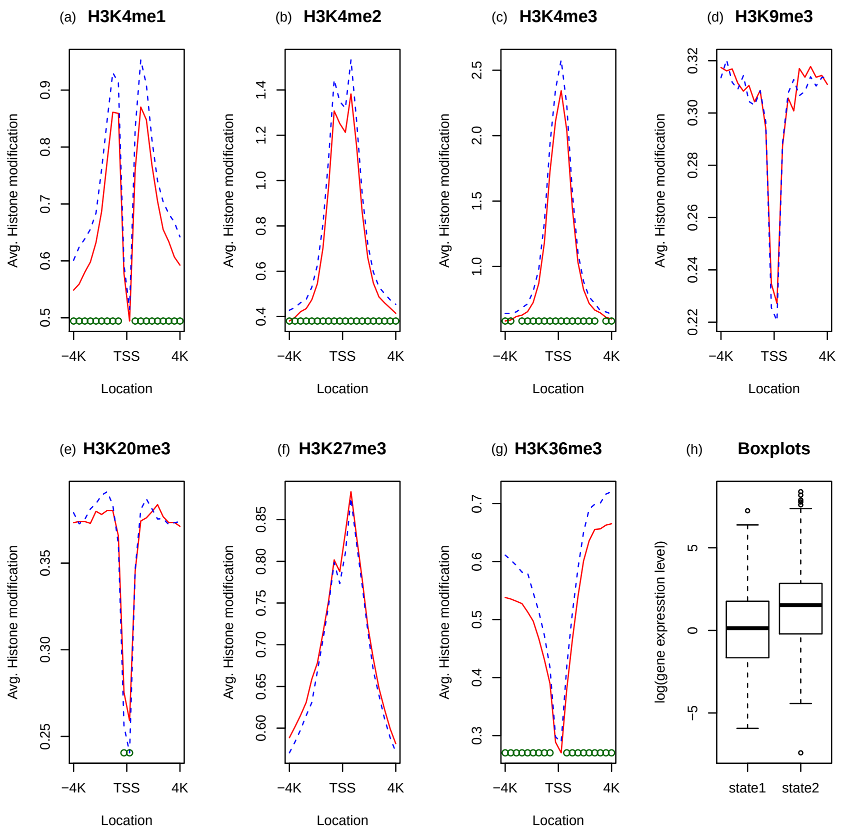

Figure A3 Average histone modification levels for each state on TSS ± 4Kb The plots (a) - (g) show the average histone modification levels in 400 bp non-overlapping windows on TSS ± 4Kb region for each state. The green circles at the bottom indicate variables of which averages are statistically significantly different from 0 after the Bonferroni correction.The box plots in (h) show the gene expression levels for each state. The gene expression level averages are 0.13 and 1.35 and the medians are 0.13 and 1.53, respectively, in State 1 and State 2.

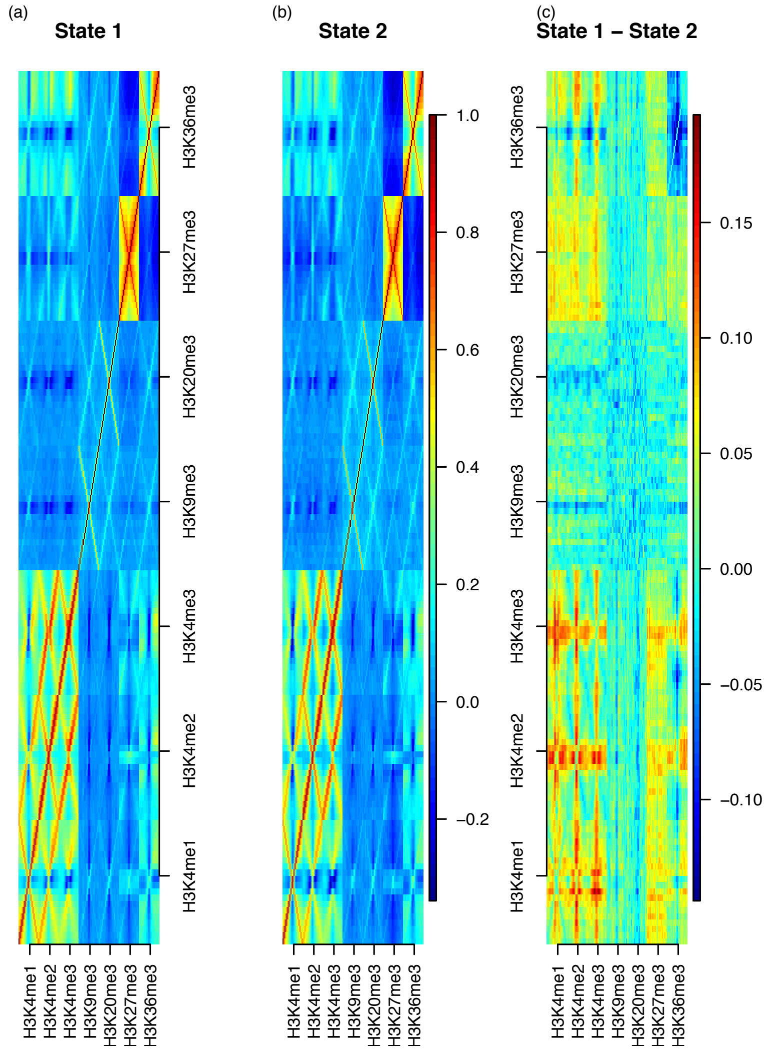

Figure A4 Correlation of the histone modification levels for each state and the difference on TSS $\pm$ 4Kb The correlation of histone modification levels for each state are in (a) and (b). The difference of them (State 1 - State 2) is plotted in (c).

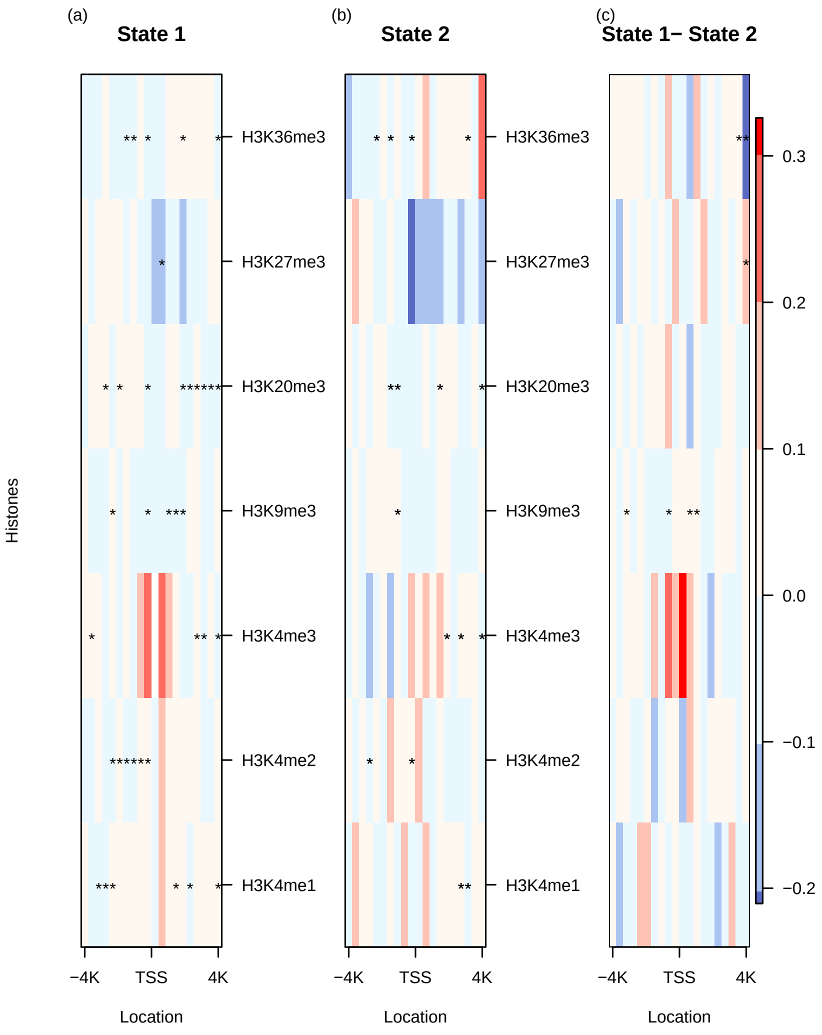

Figure A5 Regression Coefficients on TSS ± 4Kb Regression coefficients for each state are presented in (a) and (b) and the difference (State 1 - State 2) in (c). Significant coefficients after the Bonferroni correction are marked by stars. The intercepts (not shown) are .23 and 1.22 for State 1 and State 2, respectively.
