# Supplementary material for: Regression hidden Markov modeling reveals heterogeneous gene expression regulation: a case study in mouse embryonic stem cells
Source: BMC Genomics. 2014 May 12;15(1):360. doi: 10.1186/1471-2164-15-360 (PMC4144088; doi:10.1186/1471-2164-15-360)
Supplement: Supplementary file 2 — Additional file 2: A table of the more frequent GO annotations in each state. Additional file 2 contains a table describing the more frequent GO annotations in each state. The GO annotations are in bold text if the genes in the same GO annotation are better (or worse) explained by histone methylation levels in State 1 (State 2) than random genes in the annotation group of size equal to the number of genes. (PDF 39 KB) [file 12864_2013_6176_MOESM2_ESM.pdf]

Table A1: More frequent GO annotations in each state: The GO annotations are in bold text if the genes in the annotation are better or worse, in State 1 and State 2, respectively, explained by histone modification levels.

State	Functions	GO annotations	p-value	State 1	State 2	$R^2(P(r^2 < R^2))$
1	G-protein coupled receptor signaling pathway	GO:0007186	$< 10^{-16}$	549	122	0.5573 (0.121)
1	G-protein coupled receptor activity	GO:0004930	$< 10^{-16}$	448	81	0.4704 (0.0)
1	**receptor activity**	GO:0004872	$< 10^{-16}$	869	293	0.6204 (0.999)
1	signal transducer activity	GO:0004871	$< 10^{-16}$	519	143	0.6161 (0.889)
1	**integral to membrane**	GO:0016021	$1.11 \cdot 10^{-16}$	2185	1165	0.6166 (1)
1	**signal transduction**	GO:0007165	$4.44 \cdot 10^{-16}$	758	320	0.6090 (0.983)
1	extracellular region	GO:0005576	$1.21 \cdot 10^{-8}$	695	339	0.4815 (0.0)
1	response to stimulus	GO:0050896	$1.28 \cdot 10^{-7}$	80	17	
1	peptidase inhibitor activity	GO:0030414	$6.25 \cdot 10^{-6}$	57	12	
1	negative regulation of peptidase activity	GO:0010466	$2.35 \cdot 10^{-5}$	56	13	
2	**cytoplasm**	GO:0005737	$< 10^{-16}$	2137	1844	0.4753 (0.0)
2	**nucleus**	GO:0005634	$< 10^{-16}$	2002	1714	0.4971 (0.0)
2	**protein binding**	GO:0005515	$1.07 \cdot 10^{-7}$	1374	1142	0.5278 (0.029)
2	**nucleotide binding**	GO:0000166	$4.31 \cdot 10^{-7}$	779	686	0.4853 (0.0)
2	**cell cycle**	GO:0007049	$1.09 \cdot 10^{-6}$	200	220	0.4175 (0.0)
2	**cytoskeleton**	GO:0005856	$1.08 \cdot 10^{-05}$	359	340	0.4583 (0.0)
2	**apoptotic process**	GO:0006915	$1.80 \cdot 10^{-5}$	201	209	0.5566 (0.022)
2	perinuclear region of cytoplasm	GO:0048471	$2.30 \cdot 10^{-5}$	142	158	0.5971 (0.80)
2	ruffle	GO:0001726	$3.46 \cdot 10^{-5}$	18	40	
2	**ATP binding**	GO:0005524	$6.36 \cdot 10^{-5}$	550	478	0.4928 (0.0)
