# Supplementary material for: Regression hidden Markov modeling reveals heterogeneous gene expression regulation: a case study in mouse embryonic stem cells
Source: BMC Genomics. 2014 May 12;15(1):360. doi: 10.1186/1471-2164-15-360 (PMC4144088; doi:10.1186/1471-2164-15-360)
Supplement: Supplementary file 3 — Additional file 3: Analysis results for 3-state and 4-state models. Additinal file 3 contains a table describing the number of genes in each state and R 2, GO analysis results, and average histone methylation levels for 3-state and 4-state models. (PDF 123 KB) [file 12864_2013_6176_MOESM3_ESM.pdf]

SUPPLIMENTAL FILE 3

Supplemental file 3: GO Analysis and the average histone methylation levels in 3-state and 4-state models

Yeonok Lee[*†], Debashis Ghosh[†] and Yu Zhang[†]

[*]Correspondence: yul26@psu.edu
Department of Statistics, Penn State University, University Park, USA
Full list of author information is available at the end of the article
[†]Equal contributor

We extend the study to 3-state and 4-state models to see if there is more specific classification of stemness or pluripotency genes. The number of genes and R^2 in each state are presented in Table 1. GO analyses results for 3-state and 4-state models are shown in Tables and 3, respectively. The average histone methylation levels for each state for the 3-state and 4-state models are presented in Figures 1 and 2, respectively.

Overall, we found exactly 2 out of 3 (or 4) states in the 3-state (or 4-state) model that have common GO annotation enrichments with those observed in the 2 state model, whereas the rest states do not have significant GO annotation enrichment.In addition, the states with the lowest R^2 show the highest average and median gene expression levels. We suspect that this is in part due to the facts that the genes that are annotated with stemness or pluripotency may not have strong association trend with histone methylation levels.

Table A1 Number of genes and R^2 in three and four-state models

2*State	3-state model		4-state model	
	Number of genes	R^2	Number of genes	R^2
1	6163	0.73	9153	0.56
2	3491	0.73	3426	0.40
3	7366	0.38	1663	0.80
4			2778	0.78

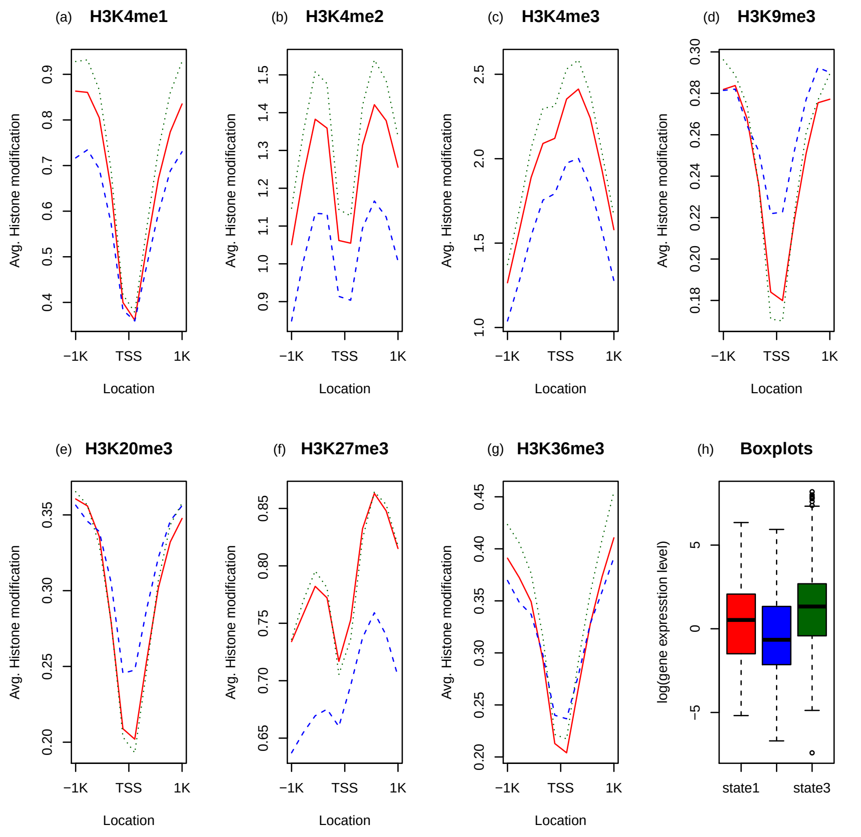

Figure A1 Average histone methylation levels for 3-state model: The plots (a) - (g) show the average histone methylation levels in 200bp non-overlapping windows on TSS $\pm$ 1Kb region for each state. The red, the blue, and the green lines represent State 1 and State 2, and State 3, respectively. The box plots in (h) show the gene expression levels for each state. The gene expression level averages are 0.40, -0.42, 1.18 and the medians are 0.53, -0.65, and 1.33 in State 1 and State 2, and State 3, respectively.

Table A2 GO Analysis : 3-state model: The GO annotations in State 2 and State 3 are in bold text if they are common in State 1 and State 2, respectively, in the two-state model (Table A1). State 1 does not have enriched GO annotations.

State	GO function	GO ID	p-value	State1	State2	State3
2	**integral to membrane**	GO:0016021	$< 2.2 \cdot 10^{-16}$	1181	879	1290
2	**G-protein coupled receptor signaling pathway**	GO:0007186	$< 2.2 \cdot 10^{-16}$	187	340	144
2	**signal transduction**	GO:0007165	$< 2.2 \cdot 10^{-16}$	351	367	360
2	**G-protein coupled receptor activity**	GO:0004930	$< 2.2 \cdot 10^{-16}$	143	285	101
2	**receptor activity**	GO:0004872	$< 2.2 \cdot 10^{-16}$	359	464	339
2	**signal transducer activity**	GO:0004871	$< 2.2 \cdot 10^{-16}$	191	302	169
2	sensory perception of smell	GO:0007608	$< 2.2 \cdot 10^{-16}$	33	246	12
2	**response to stimulus**	GO:0050896	5.69e-10	26	52	19
2	**peptidase inhibitor activity**	GO:0030414	1.87e-05	25	32	12
2	**negative regulation of peptidase activity**	GO:0010466	4.45e-05	25	31	13
2	serine-type peptidase activity	GO:0008236	6.86e-05	33	39	26
3	**cytoplasm**	GO:0005737	1.13e-09	1381	645	1955
3	**nucleus**	GO:0005634	3.67e-09	1316	572	1828
3	**protein binding**	GO:0005515	1.93e-06	857	417	1242
3	**apoptotic process**	GO:0006915	4.18e-05	119	57	234

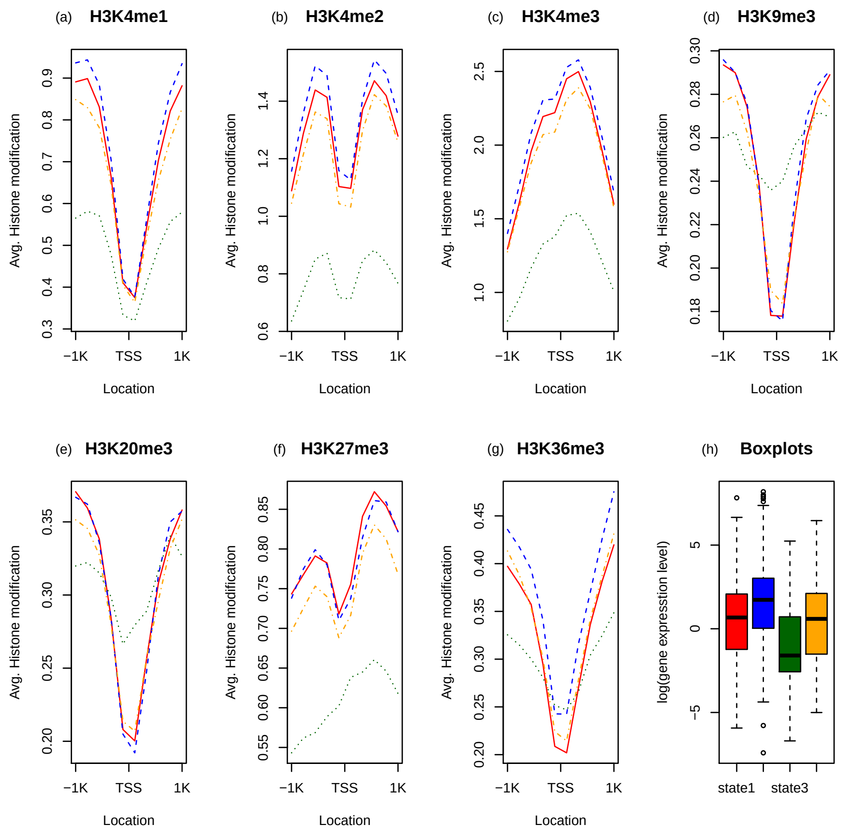

Figure A2 Average histone methylation levels for 4-state model: The plots (a) - (g) show the average histone methylation levels in 200bp non-overlapping windows on TSS $\pm$ 1Kb region for each state. The red, the blue, the green, and the yellow lines represent State 1 to State 4, respectively. The box plots in (h) show the gene expression levels for each state. The gene expression level averages are 0.54. 1.51, -0.97, and 0.59 and the medians are 0.67, 1.73, -1.59, and 0.59 in State 1 to State 4, respectively.

Table A3 GO Analysis for 4-state model: The GO annotations in State 2 and State 3 are in bold text if they are common in State 2 and State 1, respectively, in the two-state model (Table A1). State 1 and State 4 do not have enriched GO annotations.

State	GO function	GO ID	p-value	State1	State2	State3	State4
2	**nucleus**	GO:0005634	3.96e-13	2060	885	186	585
2	**cytoplasm**	GO:0005737	3.15e-08	2240	897	231	613
2	**protein binding**	GO:0005515	3.13e-06	1374	578	187	377
2	**nucleotide binding**	GO:0000166	3.09e-04	840	339	62	224
3	**integral to membrane**	GO:0016021	$< 2.2 \cdot 10^{-16}$	1824	524	492	510
3	**G-protein coupled receptor signaling pathway**	GO:0007186	$< 2.2 \cdot 10^{-16}$	275	54	264	78
3	**signal transduction**	GO:0007165	$< 2.2 \cdot 10^{-16}$	542	146	256	134
3	**G-protein coupled receptor activity**	GO:0004930	$< 2.2 \cdot 10^{-16}$	214	25	229	61
3	**receptor activity**	GO:0004872	$< 2.2 \cdot 10^{-16}$	553	132	321	156
3	**signal transducer activity**	GO:0004871	$< 2.2 \cdot 10^{-16}$	299	57	233	73
3	integral to plasma membrane	GO:0005887	5.33e-05	188	41	51	35
3	carbohydrate binding	GO:0030246	1.67e-04	49	12	22	18
